# “I was trying to look after myself, but I really wasn’t”: Understanding patient’s perspectives on risk factors for lower extremity amputations

**DOI:** 10.1186/s13047-022-00589-6

**Published:** 2022-12-12

**Authors:** Marcelle Ben chmo, Lisa Matricciani, Saravana Kumar, Kristin Graham

**Affiliations:** 1 University of South Australia Allied Health and Human Performance, University of South Australia, Adelaide, SA 5000 Australia; 2 Clinical and Health Sciences, University of South Australia, Adelaide, SA 5000 Australia

**Keywords:** Lower extremity amputation, Type 2 diabetes mellitus, Risk factors, Perspectives, Qualitative

## Abstract

**Background:**

Lower extremity amputations (LEAs) as a result of type 2 diabetes mellitus (T2DM) cause considerable morbidity, mortality, and burden on the healthcare system. LEAs are thought to be preventable, yet the rate of LEAs, particularly in Australia, has risen despite the availability of preventative healthcare services. Understanding patient’s perspectives of risk factors for LEAs may provide valuable insight into why many LEAs occur each year.

**Objective:**

The aim of this study was to explore patient’s perspectives of risk factors for LEAs as a result of T2DM.

**Methods:**

A qualitative descriptive methodology involving non-probability purposive sampling was used to recruit inpatients at a tertiary metropolitan hospital in South Australia. Semi-structured interviews were conducted, and data were transcribed verbatim. Data from the interviews were analysed using thematic analysis and the constant comparison approach.

**Results:**

A total of 15 participants shared their perspectives of risk factors for lower extremity amputations. Most (86%) of participants were male and Caucasian, with a median age of 66.4 years ranging from 44-80 years. The median duration of diabetes was 25.2 years, ranging from 12-40 years. More than half of the participants had undergone a previous amputation with 86% being unemployed or retired and 73% living in metropolitan Adelaide. Two main themes emerged: competing priorities and awareness. Finance and family care were identified as subthemes within competing priorities. While subthemes in the context of awareness related to lack of awareness of risk, experiences with health care professionals and perspectives of disease severity.

**Conclusions:**

The findings from this research indicate that addressing risk factors for LEAs for patients with T2DM require a holistic and nuanced approach which considers individual patient’s circumstances, and its influence on how risks are viewed and managed.

**Supplementary Information:**

The online version contains supplementary material available at 10.1186/s13047-022-00589-6.

## Introduction

Lower extremity amputations (LEAs) are a common complication of type 2 diabetes mellitus (T2DM) [1]. LEAs are associated with considerable morbidity, functional decline, reduced quality of life, and mortality rates of up to 74%, which is higher than several types of cancers [1]. LEAs create substantial financial burden to the health care system [2]. In Australia, diabetes-related LEAs are estimated to cost the healthcare system $48 million per year, with the total cost per amputation being $23,555 and an additional expenditure of $6,065 every year afterwards [3, 4].

Peripheral arterial disease, sensory and autonomic neuropathies, and structural foot deformities predispose patients to foot ulcers that are a precursor for LEAs [5, 6]. However, both diabetic foot ulcers and LEAs are thought to be preventable [5]. Awareness of the risk factors for amputation is believed to help guide actions and interventions so that LEAs may be delayed or avoided [7]. International recommendations suggest foot self-care practices, appropriate footwear, adequate glycaemic control, diabetes education and regular involvement with health care professionals can also be preventative [8–10]. Despite improvements in understanding of risk factors for LEAs and it’s preventable nature, Australia has experienced a 30% increase in LEAs over the last decade, with estimates expected to continue to rise alongside the growing global prevalence of T2DM [3, 11, 12].

Exploring patients’ perspectives of risk factors for LEAs may provide unique insights into why potentially preventable LEAs occur. However, few qualitative studies have been undertaken on this topic. A qualitative descriptive study of 18 participants with T2DM but no history of foot complications, identified participants had a poor understanding of foot ulcers, a common precursor of LEAs [13]. Another finding was that participants had medical beliefs that differed from medical evidence, such as walking barefoot could stimulate blood flow and assist ulcer healing [13].

While this research does shed some light on patients’ perspectives of risk factors for LEAs, given that the participants in this research had no history of a foot ulceration, nor had lived experiences of lower extremity amputation, it is unknown if such life-changing experiences would likely alter their perspectives. It is possible that such cardinal events are likely to shape individual’s perspectives [13] of risk factors for LEAs which need to be explored. A poor understanding of initial risk factors for LEAs, attributed to difficulties in communicating with health care professionals and limited access to health care services, identified in a qualitative descriptive study of 22 patients who had recently had a traumatic LEA [14]. However, participants in this study did not have a LEA as a result of T2DM altering their perspectives on risk factors for LEAs [14]. While both studies [13, 14] provide important insight, both focus on patients who have not had a LEA as a direct result of T2DM.

The aim of this study was to address this knowledge gap by exploring patient’s perspectives on risk factors for LEAs as a result of T2DM. This is of particular relevance in South Australia (SA), with one of the nation’s highest prevalence rates of LEAs as a result of diabetic foot complications [11].

## Methods

### Design

This study used a qualitative descriptive (QD) research design [15]. QD involves obtaining rich descriptive experiences from participants within a particular topic to explain the process behind the unique phenomena [15, 16]. As the focus of this research was to explore patient’s perspectives on risk factors for LEAs as a result of T2DM, QD provided the ideal methodology to underpin this research [16, 17]. The conduct and reporting of this research was informed by the consolidated criteria for reporting qualitative research (COREQ) [18].

### Participant selection

Participants were recruited over a two-month period (November-December 2020) using non-probability purposive sampling. Purposive sampling is ideal when selecting participants to best explore a desired phenomena or setting being studied [15, 18, 19]. The sample size for this study was estimated to be approximately 12 - 15 participants. This estimation was based on feasibility, time, and resources, and was comparable to similar studies in this area [14, 20] as well as data saturation (when data collection gathered no new information).

### Selection criteria

Patients with a history of T2DM and a LEA who were admitted to a tertiary metropolitan hospital in SA for diabetes related foot complications were invited to take part in the study. For participants to be included in this study, they had to have a LEA as a result of T2DM and be willing and able to provide verbal and written consent. Participants who were not fluent in verbal and written English and/or had a cognitive impairment were excluded. Prior to research commencement, ethics approval for this study was obtained from the Central Adelaide Local Health Network Human Research Ethics Committee (CALHN HREC), protocol 13611 and the Human Research Ethics Committee, University of South Australia, protocol 203444.

### Participant recruitment

Initial screening for eligibility was undertaken by senior podiatrists at the tertiary metropolitan hospital in SA, who provided patients with an information sheet and gained verbal consent from the patient to be contacted by the principal researcher (M.B). The principal researcher then formally invited the patient to take part in the study and both written and verbal consent was obtained. This process was put in place to ensure the decision by the patient to participate was independent of the treating health care professional and avoid coercion. While the initial estimation for sample size was approximately 12 - 15 participants, recruitment continued to ensure no new information was generated through ongoing data collection process.

### Data collection

Participants undertook an individual in-depth, semi-structured interview. This method of data collection was chosen to gain an understanding of participant’s point of view [16, 17, 20]. Semi-structured interviews, which use open-ended questions provide opportunities to gain meaningful, insightful and deeper understandings of phenomena where observational data cannot [16]. An interview guide was developed based on the input of the research team, practising podiatrists with a special interest in the diabetic foot as well as reviewing the literature, Appendix 1. An interview protocol was established and piloted with the research team and to provide the principal researcher with interview practice. The main focus of the interview questions was related to the patient’s perspectives on risk factors for LEAs as a result of complications from the diabetic foot. The semi-structured style allowed the interviewer to request additional information for greater exploration of responses [18]. All interviews were conducted at the metropolitan hospital in the patients' private room face-to-face and were approximately 45 minutes in duration. The principal researcher who conducted the interviews, was an honours podiatry student and had no prior involvement in the patient’s care. The interviewer had had some clinical experience and had studied the diabetic foot and its management. All interviews were audio recorded along with written field notes, providing context to each interview.

### Data analysis

Data from the semi-structured interview were transcribed verbatim by an independent transcription company and a selection of the transcripts (20%) was transcribed independently by the principal researcher. The data were managed using the QSR-NVivo software ^TM^ (Version 12) and were subsequently subjected to thematic analysis using the constant comparison approach [15, 18, 19]. Thematic analysis is a recognised approach which allows for rich descriptive data to be summarised while still maintaining its depth [16, 17]. An inductive approach to thematic analysis was chosen in order to ensure the themes were derived from the interview data (as opposed to using predetermined themes used in deductive approach). By inductively identifying themes, conclusions may be drawn about participants perspectives of risk factors for LEAs [ 16, 17]. Each interview transcript was read independently by the principal researcher and ideas generated were categorised to form themes. Multiple transcripts were also coded by the supervisors to ensure consistency and reoccurring themes were developed through repeated reading of and immersion in each transcript [20]. All disagreements between the coders were resolved through further discussions.

### Trustworthiness and credibility

Interview questions were piloted by the principal researcher to ensure adequate coverage of the issues of interest. The principal researcher also participated in interview training with the supervisors prior to collecting data, providing the principal researcher with practice and ensuring that the data collection was credible and dependable [20]. The interview was conducted with participants who had no personal nor professional relationship with the principal researcher. Interview questions aimed for the participants accounts to have rich descriptions which may help to minimise any bias by the researcher [20]. During the first three interviews, the primary supervisor joined the principal researcher to act as an independent observer and provide opportunities for peer debriefing and reflexivity (self-awareness of the research team throughout the research process, constant questioning about researchers own beliefs and judgements and use of field notes after the conduct of interviews). The use of field notes, multiple coders for some interviews, peer debriefing, adherence to semi-structured interview guide, transcribing verbatim by an external transcription company also promotes triangulation, reflexivity, credibility and confirmability [17, 19, 20]. All data were de-identified to promote trustworthiness of the analysis process [20].

## Results

Eighteen inpatients at the SA tertiary metropolitan hospital admitted for diabetes-related foot complications and had a history of a LEA agreed to participate in the research project. Of the 18 participants, three were excluded due to either a family member or a partner being present in the interview and speaking for the participant, not truly reflecting the patient’s perspectives, resulting in a final sample of 15 participants. Participant characteristics are reported in Table 1. Most (86%) participants were male and Caucasian, with a median age of 66.4 years ranging from 44-80 years. The median duration of diabetes was 25.2 years, ranging from 12-40 years. More than half of the participants had undergone a previous amputation with 86% being unemployed or retired and 73% living in metropolitan Adelaide.

**Table 1:** Participant characteristics

Participant ID	Gender	Age (years)	Ethnicity	Working status	Residential Location in SA^a^	Duration of T2DM (Years)	**Number of LEA’s**
P1	Female	53	Caucasian	Employed	Metropolitan	12	1
P2	Male	78	Caucasian	Retired	Metropolitan	40	2
P3	Male	59	Caucasian	Retired	Metropolitan	20	2
P4	Male	80	Caucasian	Retired	Metropolitan	15	2
P5	Female	44	Caucasian	Unemployed	Metropolitan	26	1
P6	Male	65	Caucasian	Retired	Metropolitan	15	1
P7	Male	57	Caucasian	Employed	Metropolitan	32	2
P8	Male	76	Caucasian	Retired	Rural	32	1
P9	Male	65	Caucasian	Retired	Rural	25	2
P10	Male	82	Caucasian	Retired	Metropolitan	20	1
P11	Male	63	Caucasian	Retired	Rural	35	1
P12	Male	72	Aboriginal	Retired	Rural	40	2
P13	Male	72	Caucasian	Retired	Metropolitan	30	1
P14	Male	71	Aboriginal	Retired	Metropolitan	10	1
P15	Male	59	Caucasian	Retired	Metropolitan	26	2

^a^Rural was defined as any residential location included in the Australian bureau of section of state, category two (bounded locality) and three (rural balance) [41]

Analysis of the data identified two overarching themes, each with subthemes:

- Theme 1- “competing priorities” with the subthemes – “finance” and “family care”
- Theme 2- “awareness” with the subtheme - “lack of awareness of risk”, “experiences with health care professionals”.

### Theme 1: Competing priorities

The competing priorities theme related to participants perceived view that prioritising others (such as family care) and day to day living (such as finances) meant they neglected their own health and self-care.

### Sub-theme 1: Family care

Whilst the majority of participants recognised their family as a support, participants caring for family members with an illness, high care, or special needs often prioritised caring duties than their own health:

P3: ‘*… this was at the time that my poor wife got diagnosed with breast cancer. So, everything happened at once. I was looking after my wife, I was trying to look after myself, but I really wasn't.’*

Participants were often aware they were making the choice to prioritise others needs at the expense of their own health:

*P5: ‘My husband's unwell, we have a child with allergies... I know everybody says, don't put yourself last because who is going to look after them if you're not there. But sometimes my health got neglected because I had other people in the equation to make sure were better than I was.’*

*P1: ‘Not managing my sugars properly…being, a full-time mom to a 13-year-old autistic son, full time working, just eating whatever whenever.’*

Low social support resulted in participants feeling inevitability and helplessness about the lack of support in their family caring role impacting their ability to prioritise their health. One participant stated:

*P5: ‘It’s just the way life is, unfortunately. We've got no family, we've got no support, so it's just us two and our daughter. So that impacts the diabetes as well and I can only do what I can do.’*

### Sub-theme 2: Finance

The competing priority of finance related to challenges accessing health care. Participants concerns with finance or employment, influenced delays in seeking healthcare. Despite diabetes medication in Australia being subsidised by the government, a participant described having to go without their insulin:

*P5: ‘…Financially, we were stuck between my disability pension and, well, tax a and tax b, but we still have bills…that stress sort of put us into having to go without insulin…I had to go without sometimes which affected my diabetes.’*

Similarly, another participant described having to without food which affected their diabetes:

*P1:* ‘*You've got to choose to feed your child and put a roof over their head… so I had to go without food sometimes and this had an effect on my diabetes.’*Participants recognised financial income provided by employment as important in accessing healthcare, however, this did not translate into prioritising their diabetes care. One participant stated:

*P7: ‘So, she said, "You better go out, out to the hospital." I said, "Well, I'll go up there, but I've got to go and get the guys started at work first." So, I went to work first because I need money to look after myself and diabetes…then later on went back up to the hospital.’*

Another participant reported that, despite the need for medical care, due to the financial costs associated with seeking health care during public holidays, they sought to delay their care.

*P8: ‘It would have cost me $600 to get to the doctors then the hospital... I just didn't have that money. So, I waited till the Monday, put up with it for three days, until I saw my doctor.’*

### Theme 2: Awareness

Awareness theme related to participant’s self-reported knowledge and understanding about their risk of requiring a LEA. The median length of diabetic diagnosis was 26 years and 53% of patients had a previous amputation, yet most perceived their risks as not serious.

### Sub-theme 1: Lack of awareness of risk

It appeared participants were unaware of some risk factors such as smoking and drinking:

*P4:* ‘*Beer, it doesn't have any effect on my diabetes with the sugar count or anything like that. I can have three or four of them on a night and get up next morning and still be down the 5.8 to 6’.*

*P8:* ‘*I didn't realize until I gave it up that I was a pretty heavy smoker. Yeah. I'd smoke 15 a day, no worries.’*

Few participants acknowledged that they had been educated on risk factors but did not comply with the advice they had received to potentially avoid foot complications. This can be best illustrated by the following quotes from patients who despite being educated about avoiding injuries and not walking barefoot, continued to do so.

*P6: ‘I’ve been trying to get in the habit of making sure I wear shoes... because I walk around home and wooden floors, bad for my feet... I've been trying to make sure that I'm wearing slippers usually. Going out the back, I'm usually just wearing slides.’*

*P13: ‘I've usually got my feet covered, anyway. When I'm outside, I like to wear thongs. I'll go to the shop, covered shoes…walk around, keeping my diabetes under control. Well, I'll more or less walk it out and see if I can get it that way, you know?’*

Additionally, despite a history of diabetic foot complications such as ulcers and consequently amputations, participants seemed oblivious to all the risk factors involved in their amputation:

*I: What was the reason you needed the amputation this time?*

*P12: ‘Sugar. Poison in my bones and blood and skin.’*

*I: Can you tell me about why you think you needed an amputation?*

*P15: ‘Uh from diabetes and that’s it.’*

### Sub-theme 2: Experiences with health care professionals

Participants reported varied experiences when dealing with health care professionals in their everyday life as part of routine diabetic management. While some were unaware of the purpose of their treatment and its impact on their diabetic foot management, others were critical of lack of education from these health care professionals.

*P11: ‘They did nothing (Podiatrist), poked and prodded me… as I say, I've had no education as far as podiatry goes.’*

*P2: ‘Well not really [explain diabetes]. The main thing was just, you know, trimming the toe nails up and checking the feeling. There’s was nothing dramatic happening.’*

*P10: ‘A podiatrist doesn’t do like a lot. I go there, they soak my feet, in bubbly water. Then they dry them, and they trim my toenails…but I couldn't see that doing a lot of good.’*

*P6: ‘I don't think they were qualified [podiatrist]. They were, they were more of a, uh, making your feet look better.’*

Even when health care professionals were taking advanced treatment pathways such as hospitalisation, some participants reported no education about their diabetes.

*I:’ How did your GP talk to you about your diabetes?’*

*P11: ‘Um, not really. She, was just filling in some forms and chucked me straight in the hospital.’*

For some participants the education message conveyed by the health care professional was that they were managing their diabetes well. This had the effect of participants perceiving they were not at risk:

*P15: ‘Invariably, that's what they say "well, you're doing all the right things, just keep doing what you're doing." So, if you go back the next time and they say the same thing... you sort of lose the point of it. In fact, the only reason I went to see a dietitian in the end was to keep somebody in a job.’*

Some participants perceived that their health care professionals were exaggerating the seriousness and were interested in financial gain:

*P9: ‘I didn’t think diabetes was as serious as they said, I just thought they were trying to drum up business for themselves.’*

*P10: ‘The podiatrist didn’t explain how the diabetes affects my feet, they are more interested in selling what products they had. So, I didn’t really go there’.*

### Sub theme 3: perspectives of disease severity

Some participants perceived that once they had recovered from an amputation their risk was reduced.

*P7: ‘After the next amputation I thought, you'd better watch yourself here, you know. Even then I still sort of thought I'm okay, it's only me toes. So, I went back to having a couple of beers.’*

*P14: ‘Until then, I'd never had any problems. even after that, I never had many problems. Never went near a doctor... for years and years and years.’*

*P:15 ‘I was conscious of the fact, that it went a couple years, and nothing happened, I was okay. I was fit and healthy, except for a missing toe and with one toe missing it didn't matter.’*

For some, diabetes was perceived as a less serious health concern, compared to other commonly occurring health issues resulting in less priority.

*P6: ‘… I mean, you talk about diabetes, it's not the same as talking about heart problems or cancers. Diabetes seems to be a lot less to worry about.’*

*P8: ‘I didn't think diabetes was as serious…uh, you know compared to cancer.’*

However, some participants did acknowledge how they had mistakenly perceived that diabetes and the risk associated with LEAs was confined to “older age”.

*P5: Like older age, that’s when you’re looking down the barrel, then being 44 and now I’ve lost toes...’*

*P9: ‘Just stupidly thought that feet problems with diabetes happens when you're in your 60s and 70s.’*

## Discussion

As the prevalence of LEAs in T2DM continues to increase, it is imperative to gain firsthand accounts of patient perspectives of risk factors for LEAs [2, 3]. Findings from this research suggest that there are a range of factors that seemingly confound a patient’s ability to manage their risk of LEAs. These factors extend beyond the biological and modifiable risk factors such as glycemic index, peripheral arterial disease, neuropathy and structural foot deformities and reflect the complexities that underpin what patients encounter in their everyday lives [6]. Therefore, a holistic approach which recognizes and caters to these complexities may be required in order to better manage the risks associated with LEAs.

### Competing priorities

Competing priorities, including family care and finances, emerged as key themes that influenced participants’ perspectives on risk factors for LEAs. Competing priorities have been reported previously in a systematic review of support needs for patients with COPD, which identified finance, work, and housing as important priorties [21]. While caregiver burden has long been recognized as a phenomenon in the management of chronic disease and disability [22]. The results of the current study support previous acknowledgements the burden of caregiver roles of patients who suffer with chronic disease remains largely overlooked by health care professionals [23].

Competing priorities were particularly experienced by participants who had low social support. Participants described this as feelings of inevitability, helplessness, and ultimately inability to manage their diabetes or risk factors. Low social support from friends and family is an intangible factor [24] that can impact morbidity and mortality with the magnitude of the effect being similar to obesity, smoking, hypertension, and physical activity [25]. Our findings reflect studies that low social support networks are directly related to illness and mortality [24, 25].

Low financial resources similarly related to challenges accessing health care. Previous studies have reported that poor access to diabetes care increased the risk of LEAs [26]. In Australia, access to health care and medication is heavily subsidized by the government, however we found that low financial resources were still a barrier. Even where participants acknowledged they had access to health care, factors such as employment responsibilities impacted this. Reflecting Maslow’s hierarchy of needs that unless patients can meet their base needs such as physical and physiological safety for both themselves and their families, they may not be in a psychological position to prioritise other factors impacting their health such as accessing health care [21, 27].

### Awareness

Another theme identified in this research was participants lack of awareness. Some participants were unaware of their risks, despite having had a previous LEA and a history of diabetes for more than 25 years. This is of concern, as a health care professional may assume that long duration of chronic diseases is associated with better awareness about risk factors. One possible explanation may be that patients are receiving health information that does not cater to their current understanding. This has been supported with previous literature, highlighting that unless information is contextualized it doesn’t have an impact, which will influence engagement in preventative practices [28–30]. Ausubel’s theory, which is based on the principle that the most important factor influencing learning is what the learner already knows [31], suggests a lack of awareness of risk factors for LEAs may be in part due to ineffective education strategies.

Awareness was also influenced by experiences with health care professionals, particularly issues with patient-provider communication. The findings from the current research reflect the notion that satisfaction with patient-provider communication may be associated with greater adherence to self-management practices [32]. In a meta-analysis, the non-adherence percentage was 19% higher in those patients who reported poor patient-provider communication [33]. This supports patient centered communication theories which focus on strategies that accommodate to patient’s communication styles, may lead to improved disease outcomes through increased awareness of risk factors for LEAs [34].

Of interest, even though 53% of participants had experienced several amputations, many downplayed the severity of their diabetes and risk of further LEAs. One possible explanation for this finding is that whilst T2DM is very common, mortality rates and complications are not as commonly discussed [1, 6]. Robbins and associates [1] demonstrates that a diagnosis of cancer is perceived as a more life-threatening diagnosis than diabetes. However, the five-year mortality rates for patients with LEAs are higher than breast and prostate cancer being 46.2-56.6% compared to 31.0% for all reported cancer [35]. This follows the transtheoretical model that the focus is not to intimidate patients to change but rather to engage self-motivation and alter patient perspectives on components such as risk factors to attain self-involvement [21].

### Clinical relevance

Health care professionals may recognize risk as arising from the patients’ health status rather than in conjunction with other factors that influence risk [36]. This supports the medical model of health, where there is an optimal level of physiological functioning and below this level could be assumed as poor health [37]. Whereas the findings from the current study suggest that factors such as competing priorities may also need to be considered.

The World Health Organization (WHO) defines health as ‘a state of complete physical, mental and social well-being and not merely the absence of disease or infirmity’ [38]. Suggesting that health is multifactorial and a biopsychosocial approach is needed in clinical practice [37]. Such an approach has gained traction in recent times with the American Diabetes Association releasing a position statement on the psychosocial care of people with diabetes [39].

The findings of the current study support that a holistic approach, where intervention strategies recognize and cater to complexities such as individuals social roles and responsibilities and the need to meet basic physical and psychological needs, could improve clinical outcomes. By recognizing that patients' risk factors for a lower extremity amputation go beyond biological factors, clinicians could implement early referrals, prioritize goals, establish realistic expectations based on the patients' current priorities and alter management plans to ultimately prevent LEA’s.

The findings of the current study also provide support for the ongoing evaluation of individuals diabetes self-care competence [39]. Individual’s awareness of their condition might not always be considered as a risk factor for diabetic foot complications and hence may not be a focus for both the health professional and patient. International guidelines of the diabetic foot engourage ongoing education in a diabetic foot risk assessment, however, their current knowledge may not be assessed [8]. Assessing a patients' awareness and communication styles may allow for the adoption of strategies that enable the assimilation of learning into existing knowledge and understanding [31].

Our findings are consistent with a patient-centered approach to care, which acknowledges the importance of the patient-practitioner relationship and communication [40]. This approach aims to consider individuals psychosocial factors and surrounding social environment to develop a shared understanding of goals and barriers to treatment [40]. If clinicians consider incorporating strategies to address patients' communication needs across the continuum, including determining goals and barriers to care, effective care enviroments, and establishing the patients' needs to communicate, it may help improve both the patients and the practitioners awarness of that individuals risk factors for LEAs.

### Limitations

As with any research, there are limitations that need to be acknowledged. Firstly, this research was a qualitative study, conducted within one tertiary hospital setting. While this may limit the transferability of the findings, they do provide first-hand perspectives of patients admitted for a LEA as a result of T2DM. Another limitation to conducting interviews within a hospital setting was interruptions during the interviews. Other health care professionals needed to provide patient care during the interview, however the interview was able to be paused and then resumed. Finally, while this research provides rich information from the patient perspective, it does not provide health care professionals perspective. Further research with this stakeholder group will add to the knowledge base in this field.

## Conclusion

Collectively, this study identifies risk factors that extend beyond the biological and modifiable risk factors for LEAs. Addressing risk factors for LEA’s in patients with T2DM may require a holistic approach which considers individual patient’s circumstances, the complexities patients encounter in their everyday lives and its influence on how risks are viewed and managed. This may require challenging the status quo, and the adoption of a health care approach which considers biological, psychological, social and cultural factors. This may allow health professionals to be better equipped to identify and address factors that impede efforts to prevent LEAs. With increasing focus on reducing the impact of diabetes in the wider community, it is imperative that such patient-centered approaches are implemented.

## Supplementary Information

**Additional file 1.**

## Data Availability

The datasets analyzed during the current study are not publicly available due to confidentiality of the participants identity but are available from the corresponding author on reasonable request.

## Abbreviations

CALHN HREC: Central Adelaide Local Health Network Human Research Ethics Committee
COPD: Chronic obstructive pulmonary disease
COREQ: Consolidated criteria for reporting qualitative research
LEAs: Lower extremity amputations
QD: Qualitative descriptive
SA: South Australia
T2DM: Type 2 diabetes mellitus
UniSA: University of South Australia
