# Supplementary material for: “I was trying to look after myself, but I really wasn’t”: Understanding patient’s perspectives on risk factors for lower extremity amputations
Source: J Foot Ankle Res. 2022 Dec 12;15:89. doi: 10.1186/s13047-022-00589-6 (PMC9743707; doi:10.1186/s13047-022-00589-6)
Supplement: Supplementary file 1 — Additional file 1. [file 13047_2022_589_MOESM1_ESM.docx]

**Appendix 1: Interview Guide**

**1) Participant demographic**

o Can you tell me the reason why you’re in hospital?

o Clarify age

o Do you currently work and if so, what do you do?

o Can you tell me more about what you do on a day-day basis?

o Do you live in Adelaide or elsewhere (urban or rural)?

o Are you living with someone else or living alone? Do you receive support from family, carers?

o Can you tell me a little bit about your general health? (Probes: How long have you had type 2 diabetes? Do you smoke, drink?)

o Is this your first amputation?

**2) Perspectives of participants amputation**

o Can you tell me about why you think you needed an amputation?

o Can you tell me about what you think led to you needing an amputation (probe – ulcers; what do you think caused the foot ulcer)

- Can you tell me what you think where the risk factors that led to you’re amputation?

**3) Perspectives on Self-care practices**

o Can you tell me about what foot self-care practices you do?

o Can you tell me about what might have impacted the way you look after feet and has your amputation impacted this in anyway (priority)?

**4) Perspectives on Health care professionals & Podiatrists**

o If you see a Podiatrist can you tell me a bit about why you see a Podiatrist?

o Explain what your relationship with your Podiatrist is like?

o How do you find it understanding your podiatrist and how do you think this has affected your amputation?

o Can you tell me about any other health care professionals that are involved with your diabetes and amputations?

o Can you tell me about how they (health care professional) impacted your amputation?

o What your relationship is like with your health care professional?

o What you think the education was that they provided you with?

o Your perspectives on being able to understand the education provided?
